# Heavy metal pollution simplifies microbial networks and enhances modularity during tailings primary succession: divergent assembly dynamics for bacterial and fungal communities

**DOI:** 10.3389/fmicb.2025.1566627

**Published:** 2025-07-18

**Authors:** Min Li, Jun Liu, Dan Cao, Xueyi Chen, Jiaxin Shi, Wenzhe Hu, Chunqiao Xiao, Yun Fang

**Affiliations:** ^1^Key Laboratory for Green Chemical Process of Ministry of Education, School of Environmental Ecology and Biological Engineering, Wuhan Institute of Technology, Wuhan, China; ^2^State Key Laboratory of Agricultural Microbiology, State Environmental Protection Key Laboratory of Soil Health and Green Remediation, College of Resources and Environment, Huazhong Agricultural University, Wuhan, China; ^3^School of Environment and Geography, Qingdao University, Qingdao, China

**Keywords:** heavy metal pollution, mine tailings, primary succession, soil microbial community, community assembly process

## Abstract

Microbial community play a fundamental role in primary succession of tailings ecosystems. However, the influence of heavy metal pollution on microbial interactions and assembly dynamics during this process remains poorly understood. In this study, we investigated bacterial and fungal communities in tailing soil and biological soil crusts (BSCs) undergoing primary succession under varying heavy metal pollution. By integrating microbial community profiling with measurements of soil nutrients and heavy metal concentrations, we aimed to elucidate how pollution levels shape microbial composition, co-occurrence networks, and assembly processes. Our results revealed clear differences in soil physicochemical properties, microbial diversity, community structure, and ecological interactions between low and high pollution conditions. Under high contamination, *Burkholderiales* dominated the bacterial communities, while *Saccharomycetales* and *Pleosporales* were representative among fungi. Microbial diversity decreased with increasing pollution, accompanied by simplified co-occurrence networks and increased modularity. In highly polluted environments, both bacterial and fungal communities exhibited stronger correlations with environmental factors. Interestingly, bacterial communities were more strongly associated with soil nutrient parameters, whereas fungal communities responded more closely to heavy metal concentrations. Community assembly analysis further showed a shift toward deterministic processes in bacterial communities under high pollution, while fungal assembly remained largely stochastic. These findings highlight the differential responses of bacterial and fungal communities to heavy metal stress and underscore the critical role of pollution in shaping microbial succession in tailing ecosystems. This study provides important insights into microbial ecology under environmental stress and may inform strategies for the bioremediation and management of contaminated mine lands.

## 1 Introduction

Soil microorganisms represent the most abundant and diverse form of life on Earth, playing vital roles in nutrient cycling, energy transfer, soil formation, and the establishment of sustainable plant communities (Lopez-Lozano et al., 2016). In degraded ecosystems, microbial communities are highly sensitive to environmental fluctuations, particularly to heavy metal contamination (Meisner et al., 2021; Cruz-Paredes et al., 2017; Rousk et al., 2013). Microbial responses to pollution often manifest through shifts in community structure and interaction patterns. Such changes can significantly affect biochemical processes, ultimately influencing the ecological stability of degraded habitats (Luo et al., 2022).

Mine tailings ponds, as a representative type of severely degraded ecosystem, are characterized by low nutrient availability and elevated concentrations of toxic metals. These sites also pose a major environmental risk due to acid mine drainage. Compared to more complex systems like forests or agricultural soils, tailings ecosystems harbor relatively simplified microbial communities and geochemical conditions, offering a unique model for exploring microbial adaptation to extreme environments. During primary succession—the initial phase of ecological recovery—microorganisms drive essential processes such as water retention and nutrient accumulation (Ortiz-Álvarez et al., 2018). The native development of biological soil crusts (BSCs) is particularly critical in this context, as they stabilize the surface and promote nutrient cycling, facilitating further ecological succession (Zhou et al., 2020; Weber et al., 2022). Native microbial succession in tailings ecosystems has been shown to improve soil aggregation and organic matter accumulation (Londry and Sherriff, 2005; Shu et al., 2005; Young et al., 2013). Microorganisms are thus key players in promoting the restoration of tailings and establishing self-sustaining ecosystems (Li et al., 2022; Santini et al., 2018). However, how these microbial communities assemble and interact under different levels of heavy metal stress remains poorly understood. In tailings environments, which are typically devoid of vegetation and organic matter, microbial colonization initiates a process of primary succession. Over time, microbial communities can form structured BSCs on the tailings surface. These BSCs emerge naturally in some settings, especially in arid or semi-arid conditions, and represent a more developed successional stage compared to bare tailings. We therefore interpret the transition from bare tailings to naturally formed BSCs as a proxy for early primary succession, which allows us to assess how microbial communities and their interactions evolve under the pressure of heavy metal pollution.

Unraveling the mechanisms of community assembly is a fundamental focus in microbial ecology, primarily involving the quantification of two key ecological processes: stochastic and deterministic processes (Vellend, 2010; Nemergut et al., 2013). Stochastic processes encompass phenomena such as homogenizing dispersal, its limitations, and drift (non-dominant), all of which are grounded in neutral theory (Liao et al., 2024; Wang S. et al., 2023) and emphasize the inherent unpredictability of microbial dynamics, including birth, death, immigration, and restricted dispersal (She et al., 2024). In contrast, deterministic processes are based on niche theory (Li et al., 2023) and include mechanisms such as homogeneous and variable selection. Key environmental drivers such as pH, total organic carbon (TOC), total nitrogen (TN), and heavy metal concentrations can impose strong filters on microbial community composition and function. Tailings ecosystems provide an ideal context for investigating these assembly mechanisms due to their reduced ecological complexity. While recent studies have explored bacterial and fungal assembly in tailings, most have examined only one microbial domain in isolation. For instance, deterministic processes have been found to dominate bacterial assembly in alkaline vanadium tailings (Zhang et al., 2024b), while stochastic processes have been reported as the primary drivers for fungal communities in copper and metalloid-contaminated tailings (Liu et al., 2023; Liu et al., 2022). However, the interaction patterns and assembly dynamics of bacterial and fungal microbial communities during primary succession under varying heavy metal pollution, as well as the mechanisms regulating these patterns, remain poorly understood.

In this study, we collected tailings samples and BSCs samples from the tailing primary succession and investigated the interaction patterns and assembly dynamics of microbial communities under high and low heavy metal pollution using 16S rRNA and ITS amplicon techniques. Our study aimed to (i) elucidate changes in the diversity and composition of bacterial and fungal communities, (ii) identify microbial co-occurrence patterns and their driving factors, and (iii) investigate community assembly processes during primary succession under varying heavy metal pollution.

## 2 Materials and methods

### 2.1 Sample collection

The tailing samples were collected from Dexing Copper Mine in Dexing City (24°29′15″N; 117°45′51″E) and the Yangmei Village Rare Earth Mine in Ganzhou City (24°59′40” N; 115°2′53″E), Jiangxi Province, southern of China (Supplementary Figure S1). Both sites are located in a subtropical monsoon climate zone, with average annual temperatures of 16–19°C and precipitation of 1,600–1,800 mm. At each site, six samples were collected using a five-point (quincunx) sampling method: three from bare tailings and three from areas covered by well-developed biological soil crusts (BSCs). Samples were transported to the laboratory on ice. Each sample was divided into two portions: one stored at 4°C for physicochemical and mineralogical analyses, and the other stored at −80°C for DNA extraction. Six samples were taken from each mine tailings to analyze network and community assembly of bacterial or fungal communities.

### 2.2 Soil physicochemical measurements

Soil moisture (SM) was determined gravimetrically by calculating the weight loss before and after oven drying. Soil pH was measured in a 1:2.5 (w/v) suspension of air-dried soil and distilled water using a pH meter (Abollino et al., 2002). Total nitrogen (TN) were determined using an elemental analyzer (Elementar Vario PYRO cube and Isoprime100, Germany) after sieving the sample and wrapping it in tin foil at a dosage of 50.0 mg (Wang et al., 2025). Soil organic carbon was determined using potassium dichromate and concentrated sulfuric acid external heating method (Walkley et al., 1934). The determination of ammonium nitrogen, nitrate nitrogen and nitrite nitrogen involve freeze-drying soil samples using an FD5-3 T freeze dryer (GOLD-SIM, Beijing, China), sieving through a 100 mesh sieve, and leaching with a 2 mol/L potassium chloride extraction solution at a water to soil ratio of 5:1. After shaking for 1 h, the samples were filtered and analyzed using an AA3 flow injection analyzer (Hu et al., 2024). The determination of total phosphorus, total sulfur and heavy metal content in biological soil crusts was carried out by weighing 0.2 g of powder sample that has passed through a 100 mesh sieve and dissolving it in a crucible using an electric heating plate. Then, the inductively coupled plasma-optical emission spectrometry (ICP-OES, 5110VDV, Agilent, United States) was used for measurement (Lin et al., 2024; Singh et al., 2025; Erdiwansyah Gani et al., 2025).

### 2.3 Assessment of heavy metal pollution

Three widely used heavy metal pollution indexes were applied to assess heavy metal pollution: the Geo-accumulation Index (I_geo_), the Single Pollution Index (PI), and the Nemerow Integrated Pollution Index (NIPI) (Yang et al., 2021; Ran et al., 2022; Zhao et al., 2023). The Geo-accumulation index (I_geo_) is a widely used method for quantitatively assessing the degree of heavy metal pollution in soils (Shi et al., 2023). This method not only considers the influence of natural geological processes on background values but also fully accounts for the impact of human activities. The formula for calculating I_geo_ is as follows:


(1) $$I_{\mathrm{geo}}=\log_{2}(C_{n}/1.5\;B_{n})$$

Where C_n_ represents the measured concentration of the heavy metals in the soil, Bn is the geochemical background value for the heavy metals, and 1.5 is a correction factor to account for background value fluctuations due to geological variations, thereby helping to identify anthropogenic impacts.

The Single Pollution Index (PI) is another method that evaluates individual indicators by comparing them against assessment standards, and it is commonly used to assess the pollution level of a single heavy metal(loid) in soil (Wang et al., 2020). The calculation formula is as follows:


(2) $$\mathrm{PI}=C_{i}/S_{i}$$

Where Ci is the measured concentration of the heavy metals, and Si is the environmental quality standard value. For soil, the risk screening values for agricultural land from the Soil Environmental Quality Standard (GB 15618-2018) are used.

The Nemerow Integrated Pollution Index (NIPI) has been widely used in evaluating soil quality in contaminated sites. This method comprehensively assesses the pollution levels of multiple heavy metals indicators, emphasizing the impact of high-concentration heavy metals on environmental quality (Huang et al., 2018). The formula is as follows:


(3) $$\text{NIPI}=\frac{\sqrt{{{\mathrm{PI}}_{\mathrm{ave}}}^{2}+{{\mathrm{PI}}_{\max}}^{2}}}{2}$$

Where PI_max_ represents the maximum single pollution index, and PI_ave_ is the average of all single pollution indices for the heavy metals.

### 2.4 Soil DNA extraction, 16S rRNA and ITS gene amplification and sequencing

Genomic DNA was extracted from 1 g of each sample using a modified phenol/chloroform method (Fang et al., 2015). DNA purity and concentration were assessed using a NanoDrop spectrophotometer (Thermo Scientific, United States), and integrity was checked via agarose gel electrophoresis (Boesenberg-Smith et al., 2012). The V4 region of the bacterial 16S rRNA gene was amplified using primers 515F (5’-GTGCCAGCMGCCGCGGTAA-3′) and 806R (5’-GGACTACHVGGGTWTCTAAT-3′), and the fungal ITS2 region was amplified using primers ITS1-F (5’-CTTGGTCATTTAGAGGAAGTAA-3′) and ITS2 (5’-GCTGCGTTCTTCATCGATGC-3′) (Apprill et al., 2015; Liang et al., 2020). Amplicons were sequenced on the Illumina HiSeq platform. Raw paired-end reads from Illumina sequencing were de-noised, de-replicated, filtered, and merged by Quantitative Insight into the Microbial Ecology (QIIME2) platform (2023.12) following the DADA2 pipeline (Callahan et al., 2016). A table summarizing amplified sequence variants (ASVs) from representative sequences was utilized for downstream analysis. The taxonomic identities of the bacteria were determined using RDP software (Wang et al., 2007) and Silva schemes (Quast et al., 2013).

### 2.5 Microbial network construction

Microbial networks were constructed separately for bacterial and fungal communities under different heavy metal pollution conditions according to previous method (Yang et al., 2024). To avoid erroneous correlations, bacteria select ASVs with abundance in the top 700, and fungi select ASVs with abundance in the top 300. Co-occurrence networks of bacterial and fungal communities under different heavy metal pollution were constructed based on the Pearson correlation correlations (*ρ* > 0.3, *p* < 0.05) of log-transformed ASVs abundances (Yuan et al., 2021). A set of metrics including the number of nodes, number of edges [NE], connectance, average degree [AD], global clustering coefficient [GCC], average clustering coefficient [ACC], average neighborhood [AN], centralization degree [CD], and eigenvector centralization [EC], and cohesion was calculated to describe the network. Cohesion was calculated as described by Yuan et al. (2021). In the networks, nodes represent ASVs, and edges represent Pearson associations between nodes. Interacted vertices represent co-occurrences across samples. A comprehensive index was established to reflect the complexity of microbial network (Zhang C. et al., 2024), and was calculated by averaging the standardized scores of the topological properties including the number of nodes, NE, connectance, AD, GCC, ACC, AN, CD, EC, and positive cohesion, as follows:


$$\text{Complexity}=(X_{\mathrm{raw}}-X_{\min})/(X_{\max}-X_{\min})$$

where X_raw_, X_min_, and X_max_ represent the raw topological properties, the minimum and maximum values across all plots, respectively.

### 2.6 Phylogenetic trees and community assembly processes quantified

To construct the phylogenetic tree, we generated a tree file from the obtained taxa using the “qiime phylogeny align-to-tree-mafft-fasttree” code used for subsequent *β*-Nearest Taxon Index (βNTI) analysis (Letunic and Bork, 2007). β-Nearest Taxon Index (βNTI) and RC_bray_ (Bray–Curtis-based Raup-Crick Index) were calculated using the “picante” R package. The relative importance of five ecological processes in tailings under different heavy metal pollution were determined based on the value of βNTI and RC_Bray._ In general, |βNTI| ≥ 2 and |βNTI| < 2 represent dominant determinism and stochasticity in driving microbiome assembly, respectively. Heterogeneous selection: βNTI < −2, homogeneous selection: βNTI > + 2, dispersal limitation: |βNTI| < 2 and RC_Bray_ > 0.95, homogenizing dispersal: |βNTI| < 2 and RC_Bray_ < −0.95, and undominated: |βNTI| < 2 and |RC_Bray_| < 0.95.

### 2.7 Data processing and statistical analysis

Differences in soil physicochemical properties were analyzed using one-way ANOVA followed by LSD and Waller Duncan multiple comparison tests. All analyses not explicitly stated in this study were conducted using R (version 4.1.0). Differences in heavy metal indexes between copper mine tailings and rare earth mine tailings was analyzed using Student’s *t*-test. The alpha diversity of bacterial and fungal microbial community composition was calculated using “Vegan” package (Oksanen et al., 2001). Partial Mantel tests were conducted using the “ggcor” package to examine correlations between microbial communities and environmental factors.^1^ The networks were constructed using the “igraph” package and visualized with Gephi platform.^2^ Gephi’s Louvain algorithm was applied to divide modules. Pearson correlation analysis between module abundance and environmental factors was conducted using “Hmisc” package (Harrell and Dupont, 2019). Statistical significance was defined as *p* < 0.05. The adjusted *p*-values (Benjamini Hochberg method) are denoted as *P_adj_*, and statistical significance was determined by *P_adj_* < 0.05.

## 3 Results

### 3.1 Geochemical characteristics of soil under different heavy metal pollution

According to the composite heavy metal pollution index (NIPI), heavy metal pollution in copper mine tailings was significantly higher than that in rare earth tailings (Table 1, Student’s *t*-test, *P_adj_* = 0.018). Six copper mine tailings samples, including three bare tailing samples (HT) and three BSCs samples (HB), were grouped together as “group H,” representing high heavy metal pollution. Similarly, six rare earth mine tailings samples, consisting of three bare tailing samples (LT) and three BSCs samples (LB), were categorized as “group L,” representing low heavy metal pollution. Nutrient parameters, such as total phosphorus and total sulfur, were significantly higher in group H than in group L. The BSC samples analyzed in this study represent naturally developed microbial communities that have colonized the tailing surface over time. This contrasts with the bare tailings, which lack visible biological coverage. Thus, we interpret the comparison between these two types of samples as indicative of different stages of early primary succession in the tailings environment. Relative to bare tailings, BSCs samples exhibited elevated concentrations of Fe, Cd, and Mn at both H and L groups, Zn at L group, and Al at H group, whereas Cu at H group showed a decreasing trend (Supplementary Table S1; Tukey’s HSD, *p* < 0.05). Moreover, nutrient parameters were significantly influenced by the presence of biocrusts (Supplementary Table S1), with total organic carbon (TOC), total nitrogen (TN), NH₄^+^-N, and total sulfur all increasing during succession.

**Table 1:** Heavy metal pollution indexes.

Indexes	Heavy metal	Copper mine tailings	Rare earth mine tailings	*P*	*P_adj_* ^a^
I_geo_^b^	Fe	4.15 ± 4.36	3.66 ± 4.48	0.005	0.021
	Cu	2.73 ± 5.30	−4.11 ± 5.37	<0.001	0.004
	Zn	−6.93 ± 4.55	−6.15 ± 4.70	0.017	0.051
	Cd	−5.78 ± 4.52	−6.28 ± 4.85	0.100	0.199
	Pb	−11.72 ± 1.85	−8.54 ± 3.09	0.035	0.087
	Mn	2.82 ± 4.37	2.26 ± 4.38	0.006	0.021
	Al	3.23 ± 4.70	4.31 ± 4.01	0.106	0.199
PI^c^	Fe	1.56 ± 0.08	1.12 ± 0.10	0.899	0.904
	Cu	10.87 ± 0.32	0.10 ± 0.01	0.191	0.319
	Zn	0.34 ± 0.08	0.58 ± 0.05	0.846	0.904
	Cd	1.96 ± 0.07	1.42 ± 0.33	0.904	0.904
	Pb	0.06 ± 0.03	0.52 ± 0.16	0.216	0.325
	Mn	0.55 ± 0.03	0.37 ± 0.01	0.881	0.904
	Al	1.64 ± 0.85	3.23 ± 0.99	0.778	0.904
NIPI^d^		5.93 ± 0.17	1.89 ± 0.44	0.002	0.018

^a^Significance levels are denoted as *P_adj_* < 0.05.

^b^I_geo_ was calculated using equation (1).

^c^PI was calculated using equation (2).

^d^NIPI was calculated using equation (3).

### 3.2 Variation in soil microbial community structure under different heavy metal pollution

**Figure 1:**
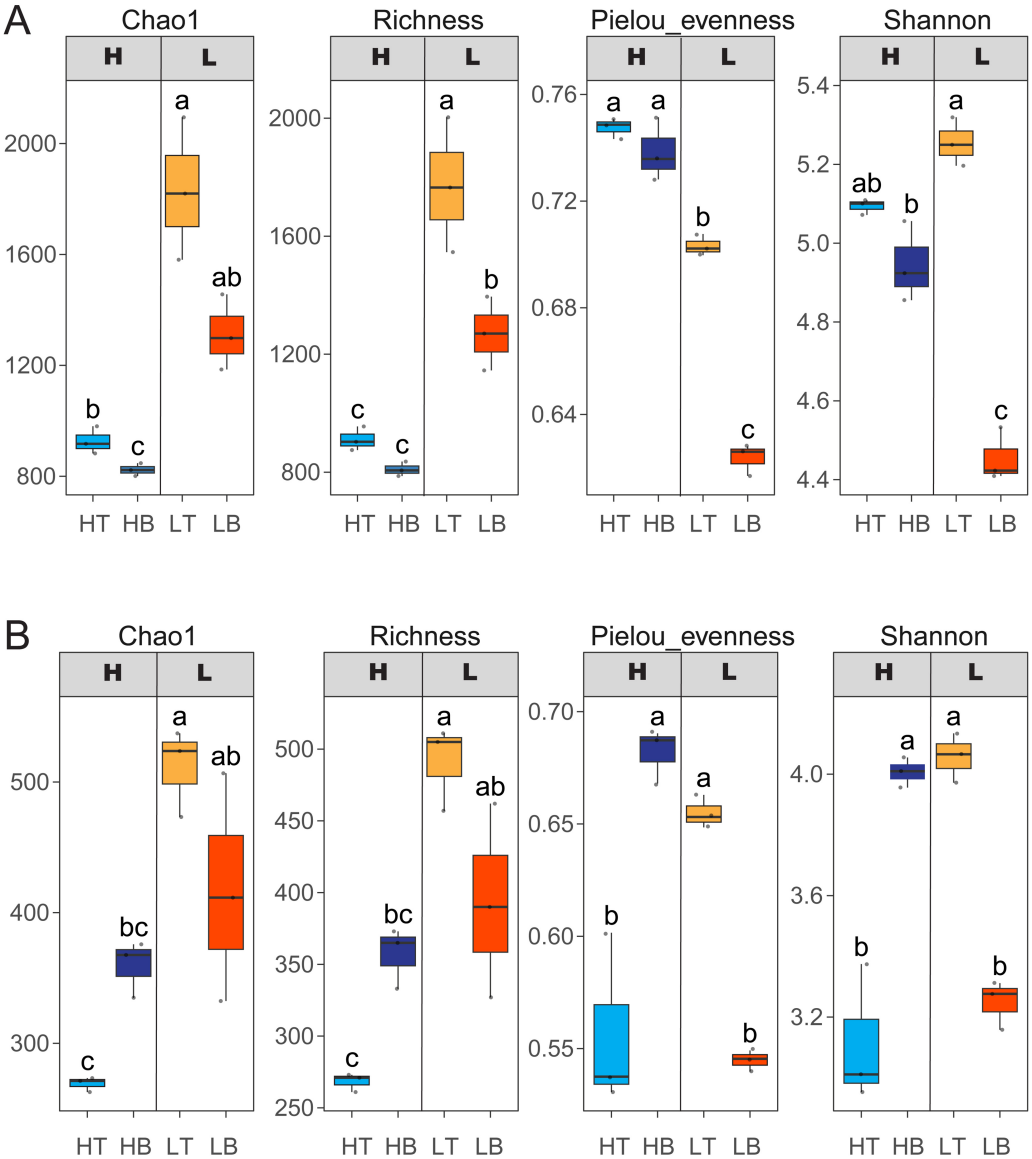
Alpha diversity of bacterial and fungal communities in soils from two types mine tailings. **(A)** Alpha diversity indices of bacterial communities. **(B)** Alpha diversity indices of fungal communities. H: Copper mine tailings; L: Rare earth mine tailings. HT: Bare tailing samples from copper mine; HB: Biological soil crust (BSC) samples from copper mine; LT: Bare tailing samples from rare earth mine; LB: BSC samples from rare earth mine.

The alpha diversity of bacteria and fungi revealed significant differences between group H and group L (Figure 1). Chao1 and Richness indices were lower in group H for both bacteria and fungi. In both groups, bacterial and fungal diversity tended to decrease with primary succession, except for fungi in group H, which showed a significant increase in all alpha-diversity metrics (Chao1, Richness, Pielou evenness, and Shannon index) after biocrust colonization. Across all samples, bacterial diversity was consistently higher than fungal diversity, suggesting a potentially more complex role of bacteria in early succession.

**Figure 2:**
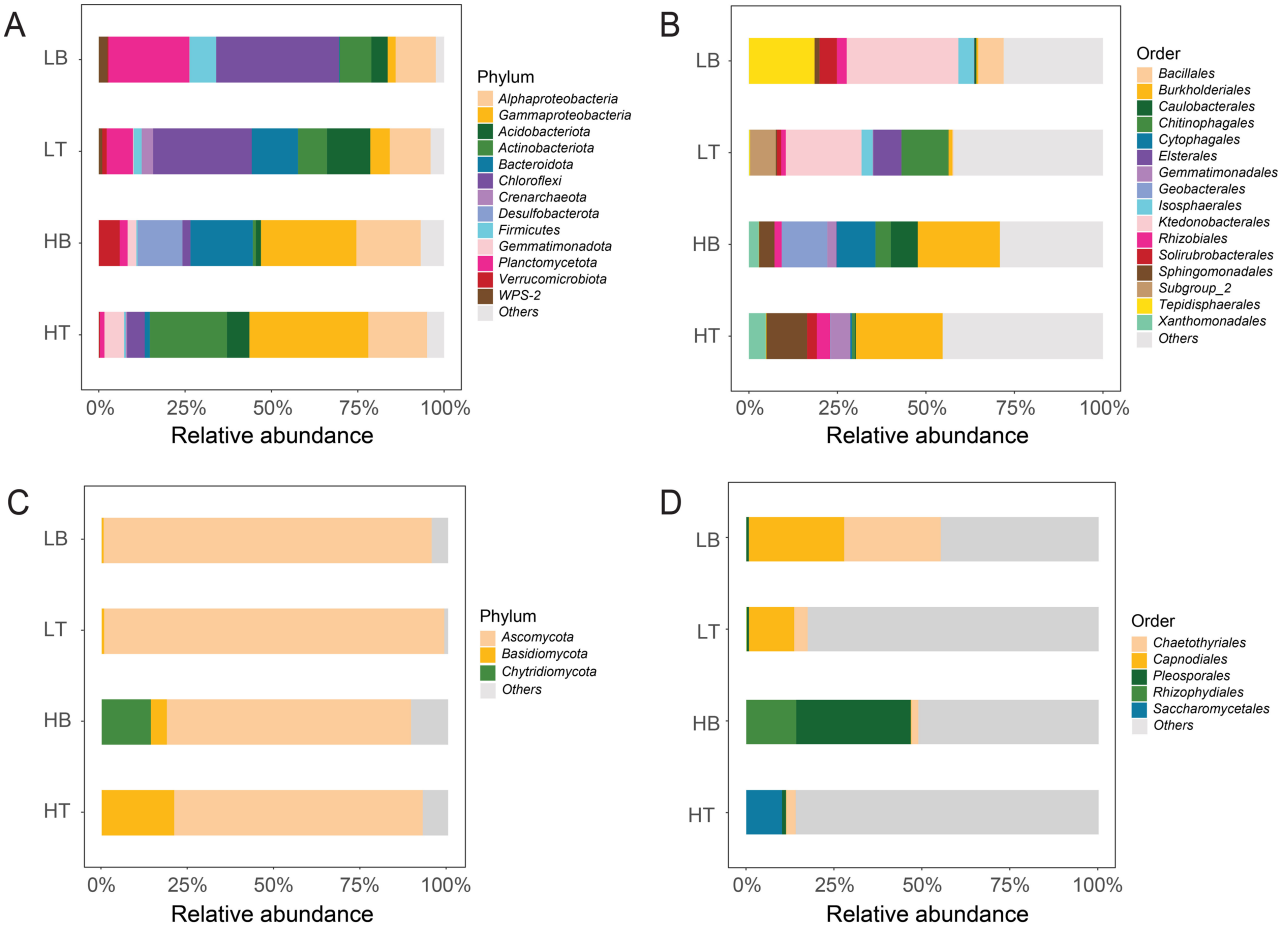
Community composition of bacterial and fungal taxa in tailing and BSC samples under different pollution levels. **(A)** Relative abundances of bacterial communities at the phylum level; **(B)** Relative abundances of bacterial communities at the order level; **(C)** Relative abundances of fungal communities at the phylum level; **(D)** Relative abundances of fungal communities at the order level. HT: Bare tailing samples from copper mine; HB: Biological soil crust (BSC) samples from copper mine; LT: Bare tailing samples from rare earth mine; LB: BSC samples from rare earth mine.

Microbial community composition also varied significantly with pollution level (Figure 2). At the phylum level, the relative abundance of *Alphaproteobacteria* (17.8 ± 1.1%), *Gammaproteobacteria* (31.0 ± 3.4%), *Gemmatimonadota* (4.1 ± 1.6%) in group H were significantly higher than that in group L (Supplementary Table S2; Tukey’s HSD, all *p* < 0.05), while the relative abundance of *Chloroflexi* (32.1 ± 3.5%), *Firmicutes* (5.0 ± 2.7%), *Planctomycetota* (15.6 ± 8.0%), and WPS-2 (1.8 ± 0.8%) in group L were significantly higher than that in group H (Supplementary Table S2; Tukey’s HSD, all *p* < 0.05). Within group H, HT samples were dominated by *Pseudomonas* (~50%), along with *Alphaproteobacteria*, *Gammaproteobacteria*, and *Actinobacterota*. HB samples also featured *Pseudomonas*, with additional contributions from *Bacteroidota* and *Desulfobacterota*. In group L, LT was enriched in *Bacteroidota*, *Chloroflexi*, *Acidobacteriota*, and *Alphaproteobacteria*, while LB primarily consisted of *Chloroflexi*, *Planctomycetota*, and *Alphaproteobacteria*. At the order level, *Burkholderiales* (23.9 ± 0.7%), *Gemmatimonadales* (4.0 ± 1.5%), and *Xanthomonadales* (3.8 ± 1.0%) were significantly more abundant in group H, whereas *Isosphaerales* (3.8 ± 0.7%) and *Ktedonobacterales* (26.7 ± 5.1%) were enriched in group L (Supplementary Tables S2, S3; Tukey’s HSD, all *p* < 0.05). *Burkholderiales* and *Sphingomonadales* dominated HT; HB featured *Burkholderiales*, *Cytophagales*, and *Geobacterales*. *Chitinophagales* and *Tepidisphaerales* were characteristic of LT and LB, respectively.

For fungi, *Ascomycota* dominated both groups (70.7 ± 2.0% in H; 94.4 ± 1.1% in L). At the order level, *Saccharomycetales* (10.1 ± 0.8%), *Pleosporales* (16.8 ± 1.1%) and *Rhizophydiales* (14.2 ± 1.3%) dominated in group H. The group HT were mainly *Saccharomycetales*, while group HB were mainly *Pleosporales* and *Rhizophydiales*. *Chaetothyriales* (20.0 ± 0.6%) and *Capnodiales* (3.3 ± 0.7%) were dominated in group L (Supplementary Tables S4, S5). Generally, the variation between group H and L was higher than that between tailings and biocrust. ADONIS analysis showed that there were no significant differences between the HT and HB, LT and LB groups (all *p* = 0.1), yet there was a significant difference (*p* = 0.004) between group H and L. Therefore, we will focus on studying the construction of soil microbial communities under different heavy metal pollution.

### 3.3 Factors affecting soil microbial communities under different heavy metal pollution

**Figure 3:**
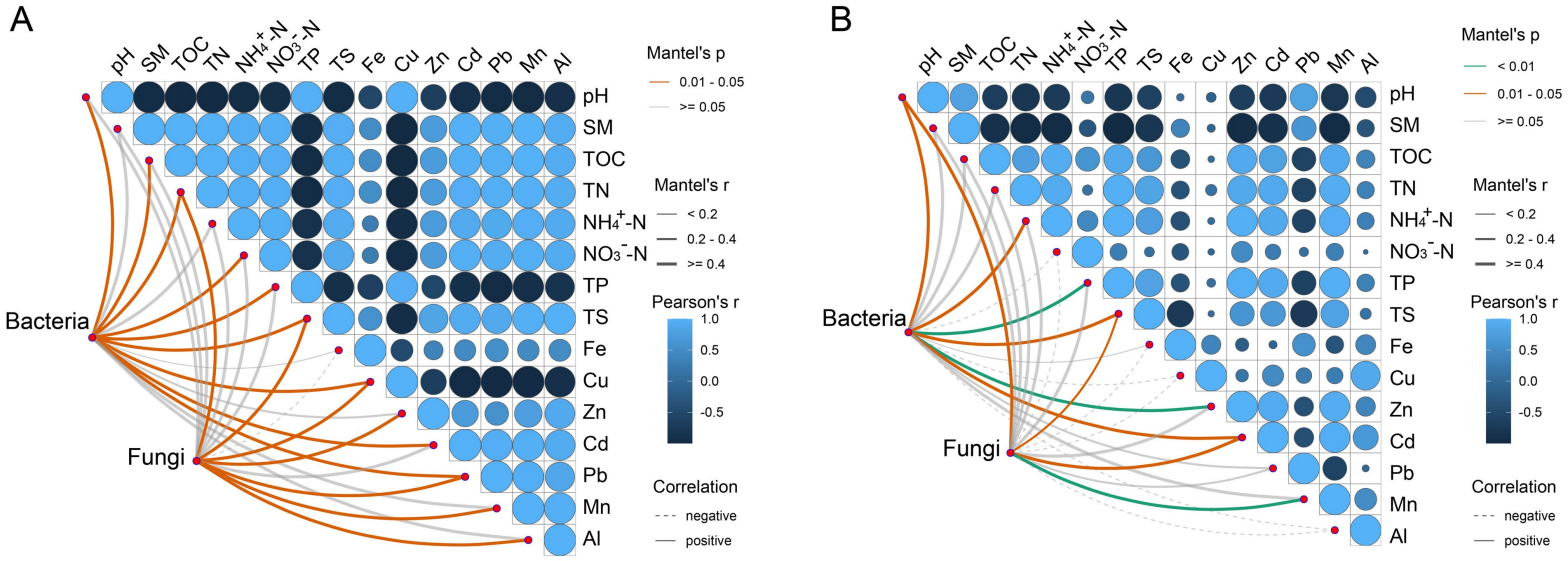
Relationships between environmental physicochemical properties and microbial community composition assessed by Mantel tests. **(A)** Mantel test results for the H group (high heavy metal pollution). **(B)** Mantel test results for the L group (low heavy metal pollution).

Mantel test analysis was conducted to investigate the correlation between environmental factors and bacterial and fungal communities in tailings under different heavy metal pollution (Figure 3). Both bacterial and fungal communities under high heavy metal pollution showed stronger correlations with environmental factors compared to those under low pollution. In group H, pH, TOC, TN, NO_3_^−^-N, TP, and TS were significantly correlated with bacterial communities (*p* < 0.05, r ≥ 0.4), while TN and TS were significantly associated with fungal communities. Metal concentrations (Cu, Zn, Pb, Mn, and Al) significantly influenced fungal communities, whereas only Cu and Pb affected bacterial communities. In group L, bacterial communities were significantly associated with both nutrients (e.g., TP) and metals (e.g., Zn), while Mn was the main driver of fungal communities. Overall, bacteria in group H were more strongly linked to nutrient variables, while fungi were more responsive to heavy metals.

### 3.4 Co-occurrence network of bacterial and fungal microbial communities under different heavy metal pollution

**Figure 4:**
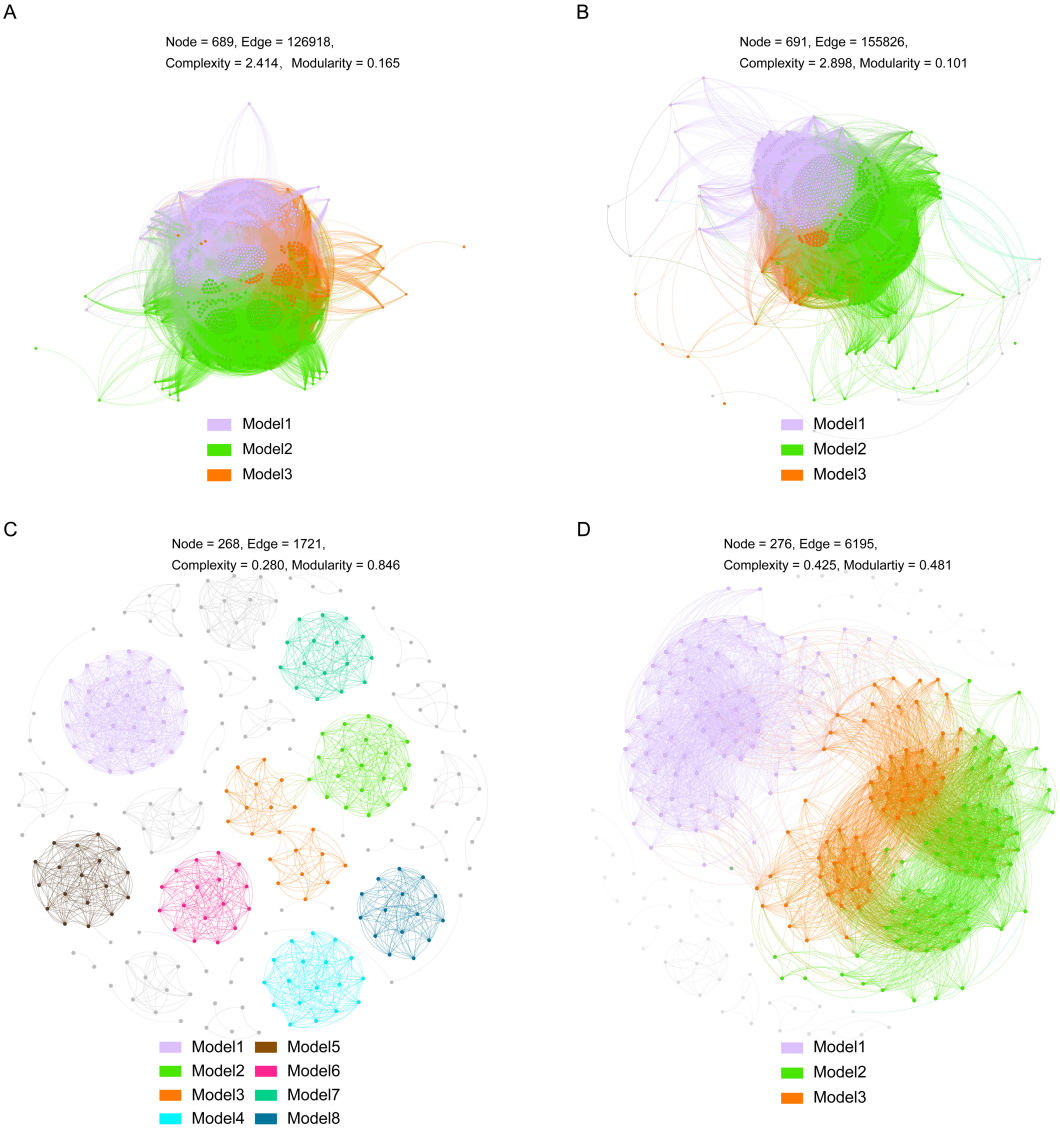
Network visualization of microbial co-occurrence patterns. **(A)** Bacterial community in copper mine tailings. **(B)** Bacterial community in rare earth mine tailings. **(C)** Fungal community in copper mine tailings. **(D)** Fungal community in rare earth mine tailings.

**Figure 5:**
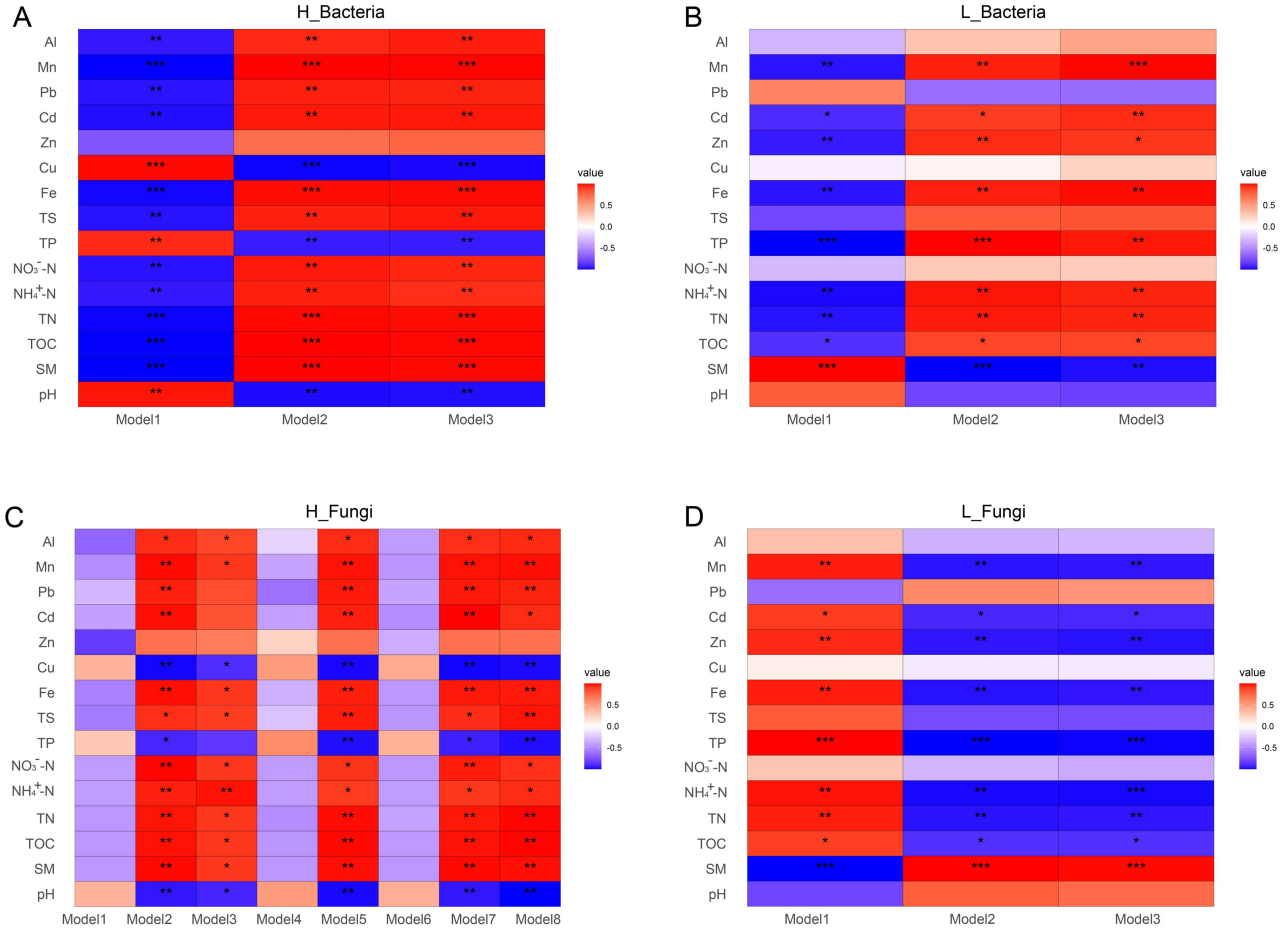
The relationships between physicochemical factors and microbial co-occurrence network modules. **(A)** Bacterial community in group H. **(B)** Bacterial community in group L. **(C)** Fungal community in group H. **(D)** Fungal community in group L. Pearson correlation analysis was performed between physicochemical factors and modules, and the *p*-values were corrected using the Benjamini-Hochberg (BH) method. Significance levels are denoted as * *P_adj_* < 0.05, ** *P_adj_* < 0.01, and *** *P_adj_* < 0.001.

To explore microbial interactions under different pollution levels, we constructed co-occurrence networks of bacteria and fungi (Figure 4). Our results indicated that these co-occurrence patterns were significantly altered under different heavy metal pollution conditions. Specifically, for both bacterial and fungal communities, network properties such as connectivity, average degree, and global clustering coefficient were lower in group H than in group L. In contrast, modularity was higher in group H compared to group L (Supplementary Table S6). Additionally, for other bacterial network properties, including average clustering coefficient and positive cohesion, values in group L were higher than those in group H. Conversely, the fungal network displayed the opposite trend. Analysis of network complexity revealed that the co-occurrence network in group H exhibited relatively low complexity. Furthermore, the bacterial and fungal networks in group H comprised 3 and 8 modules, respectively, whereas those in group L consisted of 3 and 3 modules, with nodes in modules ≥15 (Figure 4). In group H, all three bacterial modules showed significant correlations with pH, TOC, TN, and metals such as Cu, Cd, Fe, and Mn, but not with Zn (Figure 5A). Module 1 displayed correlations opposite to those of modules 2 and 3, particularly for pH, TS, and Cu (Figure 5A). In comparison to group H, the bacterial co-occurrence network of group L exhibited a weaker correlation with environmental factors. Specifically, the three modules were significantly correlated with SM, NH_4_^+^-N, and TP in nutritional factors, but did not correlate with Cu, Pb, or Al (Figure 5B). In the fungal network of group H, five of the eight modules showed significant correlations with SM, TOC, TN, Cu, and Mn (Figure 5C). In contrast, the two fungal modules in group L were only weakly correlated with most environmental variables, except SM, NH₄^+^-N, and TP (Figure 5D).

### 3.5 Assembly process of microbial communities under different heavy metal pollution

**Figure 6:**
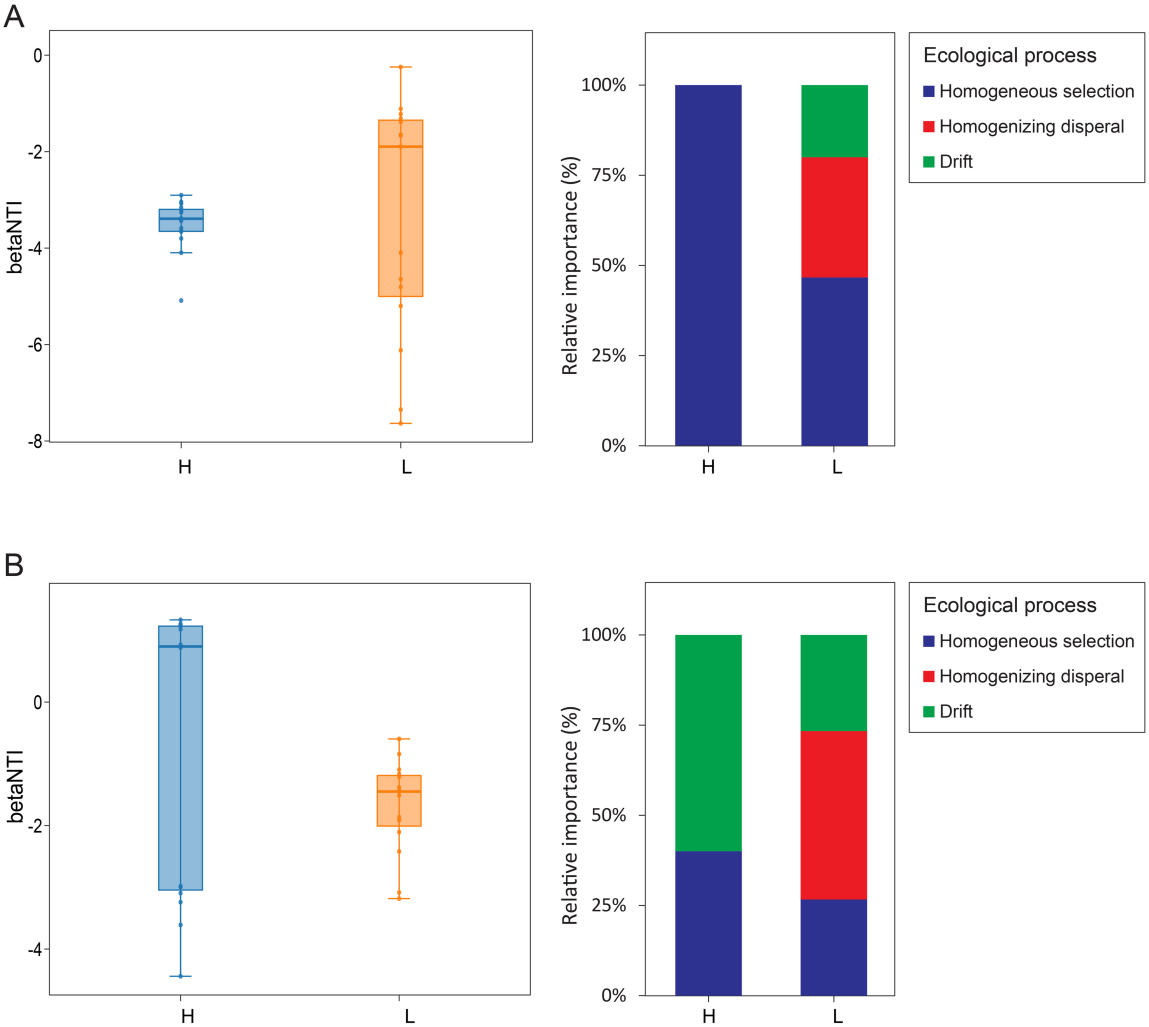
Deterministic and stochastic processes in microbiome assembly. Relative contribution of determinism and stochasticity on bacterial community **(A)** and fungal community **(B)** assembly of tailings under different pollution levels on the βNTI values and RC_Bray_ values.

The deterministic and stochastic aspects of microbial community assembly were calculated and assessed using the weighted βNTI and RCbray (Figure 6). Null model analysis revealed that the relative contributions of deterministic (|βNTI| ≥ 2) and stochastic (|βNTI| < 2) processes in bacterial and fungal communities assembly were greatly affected by different heavy metal pollution (Figure 6). In the assembly process of bacterial communities (Figure 6A), group H exhibited βNTI < −2, predominantly indicating a deterministic process driven by homogeneous selection. The |βNTI| values of Group L are partially greater than 2 and partially less than 2, reflecting the presence of heterogeneous selection, homogenizing dispersal, and drift, as inferred from the RC_Bray_ value distributions. In the assembly process of fungi communities (Figure 6B), deterministic process of heterogeneous selection and stochastic process of drifts existed in both group H and group L. Collectively, the assembly of bacterial communities in group H was primarily driven by deterministic processes, including specific environmental conditions. According to the mantle test (Figure 3), the bacterial community of copper tailings with high pollution index (Group H) is mainly affected by pH, TOC, TN, NO_3_^−^-N, TP, TS, Cu, Cd, and Pb, while the fungal community is mainly affected by TN, TS, Cu, Zn, Pb, Mn, and Al. Conversely, the assembly of fungal communities in group H, as well as both bacterial and fungal communities in group L, was influenced by a combination of deterministic and stochastic processes.

## 4 Discussion

### 4.1 Differences in microbial community composition under different heavy metal pollution

Primary succession is the process by which an ecosystem emerges from scratch and is the initial stage of ecosystem development. The primary succession that occurs on tailings faces enormous challenges from heavy metal pollution. Microbial communities, as early colonizers, play essential roles in ecosystem development but are highly sensitive to such stressors (Li et al., 2021). This study explores how heavy metal pollution shapes microbial diversity, composition, and assembly during early tailings succession. Tailings in group H had significantly higher heavy metal concentrations than group L (Table 1), indicating stronger environmental filtering. Correspondingly, both bacterial and fungal diversity, as measured by Chao1 and richness indices, were significantly lower in group H than in group L (Figure 1), suggesting that heavy metal stress suppresses overall diversity and selects for metal-tolerant taxa (Gao et al., 2021; Lima-Morales et al., 2016). Such filtering likely promotes more deterministic community assembly. Bacterial diversity declined over time in both groups (H and L), potentially due to increasing environmental specialization or competitive exclusion during biocrust formation. High diversity in bare tailings may offer functional redundancy, buffering ecological functions under extreme conditions (Li et al., 2024). Interestingly, fungal diversity increased in group H during succession, consistent with findings from reclaimed copper tailings (Zhang et al., 2016), suggesting that fungi gradually adapt and establish under metal stress. Although our study does not follow a temporal time series, the comparison between bare tailings and naturally developed BSCs offers insights into early successional dynamics in a spatial context. The distinct microbial community structures observed between the two sample types support the notion that succession is occurring, with BSCs representing a more mature stage shaped by prolonged microbial activity and environmental selection.

Community composition was also shaped by pollution levels. In group H, *Alphaproteobacteria* and *Burkholderiales* (*Gammaproteobacteria*) dominated (Figure 2A). *Alphaproteobacteria* mitigate metal toxicity by secreting organic acids and chelators (Anas et al., 2025), while *Burkholderiales* use efflux pumps to reduce intracellular metal accumulation (Wen et al., 2024). Their metabolic versatility and nitrogen fixation capacity (Qiu et al., 2023; Bellés-Sancho et al., 2023) enhance survival under nutrient-poor, metal-rich conditions. Other prevalent taxa under high stress included *Gemmatimonadales* and *Xanthomonadales*, both involved in carbon and nitrogen cycling (Whalen et al., 2024; Zhang et al., 2024a; Wang X. et al., 2023), as well as *Actinobacteria*, which are known for decomposing unstable organic carbon and producing enzymes and metabolites that enhance resistance to heavy metals (Fierer et al., 2007; Chen et al., 2021; Dinglasan et al., 2025). Additionally, autotrophic taxa such as *Chloroflexi* were detected, especially in rare earth tailings (Guo et al., 2022), contributing to ecosystem self-sufficiency through photo- and chemoautotrophy (Fernández et al., 2008).

Fungal communities showed distinct taxonomic shifts. Group H had a higher relative abundance of *Basidiomycota*, whereas *Ascomycota* dominated in group L. *Ascomycota*, mainly saprotrophs, thrive in stable environments and efficiently decompose organic matter (Ai et al., 2020). In contrast, *Basidiomycota* exhibit greater ecological adaptability, including resistance to harsh conditions through spore or hyphal structures (Pei et al., 2016; Calhim et al., 2018). At finer taxonomic levels, the abundances of *Saccharomycetales*, *Pleosporales*, and *Rhizophorales* were higher in group H compared to group L. This observation may be attributed to the inherent pollution resistance of these fungal groups, which enables them to withstand environmental pressures by forming resilient spore or hyphal structures. For instance, yeasts within the *Saccharomycetales* order can form thick-walled spores, providing resistance to heavy metal pollution (Neiman, 2005). These groups also possess diverse metabolic capabilities, enabling them to utilize a broad spectrum of substrates. *Pleosporales*, for example, are prominent decomposers capable of surviving in nutrient-poor environments (Lei et al., 2024). In group L, the competition for ecological niches among microbial communities was relatively small, and *Chaetothyriales* and *Capnodiales* may be more adapted to this relatively “mild” environment, thereby gaining a competitive advantage. We also considered analyzing microbial community composition at the genus level. However, due to the limitations of amplicon sequencing technology, it is challenging to accurately classify all ASVs at this taxonomic resolution. Specifically, 36.6% of bacterial ASVs and 80% of fungal ASVs could not be assigned to known genera. Given this limitation, we focused our analysis on the phylum and order levels, which provided more reliable and comprehensive taxonomic resolution.

Co-occurrence network analyses revealed that microbial communities under high pollution exhibited lower complexity but higher modularity (Figure 4). Heavy metal stress likely suppresses microbial biomass and activity (Chen et al., 2019), reducing overall connectivity. However, the observed increase in modularity suggests enhanced niche differentiation under resource limitation, aligning with the resource-ratio hypothesis (Kim and Ohr, 2020). Strong environmental filtering may also intensify competition among stress-tolerant taxa occupying similar niches (Fuhrman et al., 2015). Furthermore, microbial modules in group H showed stronger correlations with heavy metal concentrations (Figure 5), indicating tighter coupling between community structure and environmental variables under high stress (Li et al., 2021; Hernandez et al., 2021).

It should be noted that the co-occurrence network analysis in this study was based on a relatively small number of samples (*n* = 6) for each treatment group. While network-based approaches are useful for generating hypotheses about potential microbial interactions, a limited sample size can reduce statistical power and may lead to the overestimation of correlations among ASVs. As such, the inferred networks should be interpreted with caution, and future studies with larger sample sizes are needed to validate the robustness of the observed network patterns.

In summary, heavy metal pollution exerts strong selective pressure during primary succession in tailings, reducing microbial diversity and shifting community composition toward tolerant and metabolically versatile taxa. These changes alter microbial assembly dynamics, functional potential, and ecological interactions, ultimately influencing the trajectory of ecosystem development in metal-contaminated environments.

### 4.2 Driving factors and assembly mechanism of tailing microbial communities under different heavy metal pollution

Mine tailings represent oligotrophic environments where the early stage of ecological succession largely occurs in surface soils formed through natural accumulation. Thus, the physicochemical properties of the surface soil are critical to ecological development. The pH of tailings, influenced by beneficiation processes, carbonate content, and local soils, ranges widely from acidic to alkaline (pH 2–9) (Yang et al., 2022). The accumulation of soil nutrients carbon, nitrogen, phosphorus and sulfur was an important indicator of soil fertility and productivity (Garcia-Pichel et al., 2016). Heavy metals, in contrast, inhibit microbial activity, slow organic matter decomposition, and disrupt nutrient cycling. Mantel test results revealed that microbial communities in copper tailings under high environmental stress were more strongly correlated with environmental factors (Figure 3), consistent with previous findings (Wu et al., 2024). Hence, the intricate influence of different heavy metal pollution on soil bacterial and fungal communities stems from the simultaneous introduction of nutrients and heavy metals, resulting in complex alterations in community dynamics. Bacteria and fungi in high-pressure environments were greatly influenced by environmental factors, bacterial communities were greatly affected by nutrients, and fungal communities were greatly affected by heavy metals (Figure 3). Previous studies have shown that fungal community was significantly affected by heavy metals. Fungi were generally more sensitive to heavy metals than bacteria, exhibiting diverse responses to such contamination (Oliveira and Pampulha, 2006; Sun et al., 2024).

Understanding the community assembly mechanisms controlling the community diversity, composition, functions is a central topic of ecology (Zhou and Ning, 2017). We found that deterministic processes dominated bacterial assembly under high pollution, while both deterministic and stochastic processes shaped fungal communities. Under low pollution, assembly of both bacterial and fungal communities involved a balance of stochastic and deterministic processes (Figure 6). These findings are consistent with the hypothesis that deterministic processes increase with environmental stress (Ning et al., 2024), and align with multiple studies reporting enhanced determinism under pollution (Zhang et al., 2019; Zhang et al., 2021). Nevertheless, stochastic processes—including ecological drift and dispersal limitation—remain important across pollution gradients (Gao et al., 2020; Zhang et al., 2019; Zhang et al., 2021; Liu et al., 2022). For fungi under high pollution, both stochastic (e.g., dispersal limitation) and deterministic (e.g., environmental selection) factors were influential (Figure 6). Notably, under extreme stress, deterministic processes such as competition or filtering may weaken due to reduced fungal population sizes, amplifying the effects of drift (Bier et al., 2022). Additionally, metal stress may hinder fungal dispersal capacity, leading to dominance of dispersal limitation as a stochastic mechanism (Hawksworth and Lücking, 2017).

## 5 Conclusion

In conclusion, our study showed a significant influence of different pollution stress on various aspects including soil physicochemical parameters, heavy metal concentrations, microbial community composition, network interaction, and assembly process. Under high pollution, microbial co-occurrence networks have less complexity, but more modules, and higher modularity, and distinct modules exhibited differential responses to environmental factors. Remarkably, deterministic processes primarily governed bacterial community assembly under high heavy metal pollution, whereas stochastic processes had more influence on fungal community. Furthermore, stochastic processes, drift, play critical roles in shaping tailings microbiomes, even under high pollution conditions. These findings suggest that restoration of polluted tailings should consider strategies to overcome dispersal constraints, such as microbial inoculation. Overall, our results offer strong evidence for the influence of heavy metal pollution on microbial communities and their assembly processes. Future work should focus on microbial functional traits and ecosystem services to deepen our understanding of how microbial communities adapt to and mitigate the impacts of heavy metal pollution.

## Data Availability

The original contributions presented in the study are publicly available. This data can be found here: https://www.ncbi.nlm.nih.gov, accession number PRJNA1214178.

## Footnotes

^1^ https://github.com/houyunhuang/ggcor

^2^ http://gephi.github.io/
