# Supplementary material for: Heavy metal pollution simplifies microbial networks and enhances modularity during tailings primary succession: divergent assembly dynamics for bacterial and fungal communities
Source: Front Microbiol. 2025 Jul 18;16:1566627. doi: 10.3389/fmicb.2025.1566627 (PMC12313675; doi:10.3389/fmicb.2025.1566627)
Supplement: Supplementary file 1 [file Supplementary_file_1.docx]

**Supplementary Material**

**for**

**Heavy metal pollution simplifies microbial networks and enhances modularity during tailings primary succession: divergent assembly dynamics for bacterial and fungal communities**

**Min Li^1#^, Jun Liu^2#^, Dan Cao^2^, Xueyi Chen^1^, Jiaxin Shi^3^, Wenzhe Hu^2^, Chunqiao Xiao^1^, Yun Fang^1*^**

^1^ Key Laboratory for Green Chemical Process of Ministry of Education, School of Environmental Ecology and Biological Engineering, Wuhan Institute of Technology, Wuhan 430205, PR China;

^2^ State Key Laboratory of Agricultural Microbiology, State Environmental Protection Key Laboratory of Soil Health and Green Remediation, College of Resources and Environment, Huazhong Agricultural University, Wuhan 430070, PR China;

^3^ School of Environment and Geography, Qingdao University, Qingdao 266071, PR China;

**^#^** Min Li and Jun Liu contributed equally to this work.

**Corresponding author:** Yun Fang

E-mail: fangy@wit.edu.cn


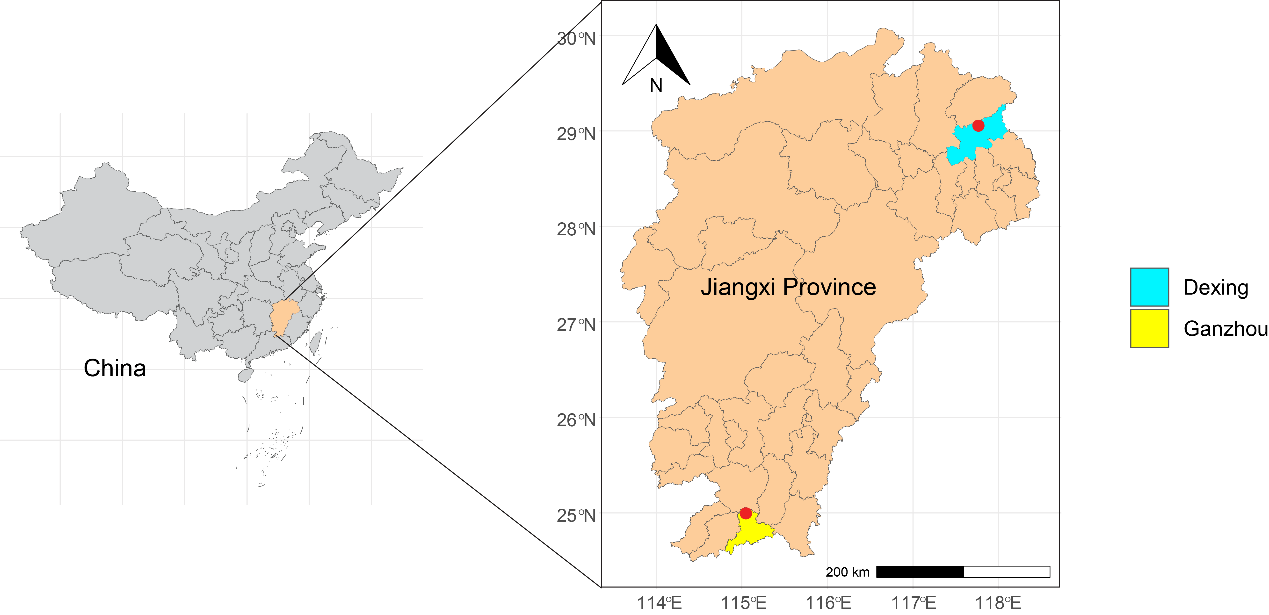


**Figure S1** Geographic location of the study area.

**Table S1** Physicochemical properties in tailings and BSCs samples

| **Samples** | **HT** | **HB** | **LT** | **LB** |
| --- | --- | --- | --- | --- |
| **pH** | 8.84 ± 0.04^a^ | 8.08 ± 0.09^b^ | 5.30 ± 0.09^c^ | 5.13 ± 0.03^c^ |
| **SM** | 0.32^d^ | 32.7^a^ | 5.24^b^ | 0.58^c^ |
| **TOC(g/kg)** | 1.21 ± 0.04^c^ | 6.79 ± 0.21^a^ | 1.35 ± 0.17^c^ | 3.83 ± 1.15^b^ |
| **TN(g/kg)** | 0.10 ± 0.01^b^ | 0.29 ± 0.01^a^ | 0.10 ± 0.00^b^ | 0.4 ± 0.047^a^ |
| **NH_4_^+^-N(mg/kg)** | 0.05 ± 0.01^c^ | 11.99 ± 2.56^a^ | 2.97 ± 0.28^c^ | 7.60 ± 0.46^b^ |
| **NO_3_^-^-N(mg/kg)** | 0.24 ± 0.02^c^ | 2.96 ± 0.46^a^ | 1.43 ± 0.37^bc^ | 1.70 ± 0.46^b^ |
| **TP(g/kg)** | 0.79 ± 0.02^a^ | 0.67 ± 0.02^b^ | 0.04 ± 0.00^d^ | 0.17 ± 0.00^c^ |
| **TS(g/kg)** | 8.46 ± 0.27^b^ | 10.28 ± 0.23^a^ | 0.44 ± 0.04^c^ | 0.53 ± 0.03^c^ |
| **Fe(g/kg)** | 31.23 ± 1.36^c^ | 44.96 ± 0.50^a^ | 22.33 ± 1.67^d^ | 38.32 ± 2.37^b^ |
| **Cu(mg/kg)** | 652.17 ± 15.47^a^ | 377.08 ± 5.53^b^ | 5.73 ± 0.68^c^ | 5.86 ± 1.02^c^ |
| **Zn(mg/kg)** | 68.23 ± 12.95^c^ | 87.25 ± 0.74^c^ | 115.42 ± 8.87^b^ | 158.46 ± 4.11^a^ |
| **Cd(mg/kg)** | 1.18 ± 0.04^bc^ | 1.97 ± 0.10^a^ | 0.85 ± 0.16^c^ | 1.53 ± 0.16^b^ |
| **Pb(mg/kg)** | 6.23 ± 2.71^c^ | 24.83 ± 1.49^bc^ | 51.61 ± 13.13^bc^ | 36.34 ± 2.12^ab^ |
| **Mn(mg/kg)** | 387.32 ± 18.81^c^ | 798.73 ± 8.17^a^ | 261.02 ± 6.90^d^ | 458.63 ± 35.50^b^ |
| **Al(g/kg)** | 32.90 ± 13.89^b^ | 93.89 ± 2.34^a^ | 64.54 ± 16.23^ab^ | 77.64 ± 21.81^ab^ |

Values were mean ± standard deviation. ANOVA followed by Tukey's test were performed. Different letters (a, b and c) in the same row indicate significant differences among samples at the level of <0.05.

HT: Bare tailing samples in cooper mine tailings; HB: BSCs samples in cooper mine tailings; LT: Bare tailing samples in rare earth mine tailings; LB: BSCs samples in rare earth mine tailings.

**Table S2** Relative abundances of major bacterial communities at phylum level of bare tailings and BSCs samples (Tukey’s HSD, *P* < 0.05).

| **Phylum** | **HT** | **HB** | **LT** | **LB** |
| --- | --- | --- | --- | --- |
| ***Alphaproteobacteria*** | 0.17031 ± 0.00391^a^ | 0.18661 ± 0.00941^a^ | 0.11841 ± 0.00432^b^ | 0.11651 ± 0.00388b |
| ***Gammaproteobacteria*** | 0.34431 ± 0.00389^a^ | 0.27631 ± 0.00512^b^ | 0.05651 ± 0.00189^c^ | 0.02311 ± 0.00091^d^ |
| ***Acidobacteriota*** | 0.06521 ± 0.00506^b^ | 0.01451 ± 0.00087^d^ | 0.12551 ± 0.00268^a^ | 0.04711 ± 0.00447c |
| ***Actinobacteriota*** | 0.22311 ± 0.00551^a^ | 0.00901 ± 0.00040^c^ | 0.08371 ± 0.00509^b^ | 0.09161 ± 0.00271b |
| ***Bacteroidota*** | 0.01481 ± 0.00029^c^ | 0.18061 ± 0.00572^a^ | 0.13381 ± 0.00513^b^ | 0.00211 ± 0.00002d |
| ***Chloroflexi*** | 0.05121 ± 0.00456^c^ | 0.02341 ± 0.00367^c^ | 0.28601 ± 0.00476^b^ | 0.35591 ± 0.00262a |
| ***Crenarchaeota*** | 0.00321 ± 0.00045^b^ | 0.00001 ± 0.00000^b^ | 0.03311 ± 0.00505^a^ | 0.00001 ± 0.00001b |
| ***Desulfobacterota*** | 0.00081 ± 0.00019^b^ | 0.12951 ± 0.00740^a^ | 0.00011 ± 0.00003^b^ | 0.00001 ± 0.00001b |
| ***Firmicutes*** | 0.00451 ± 0.00006^c^ | 0.00361 ± 0.00019^c^ | 0.02311 ± 0.00230^b^ | 0.07701 ± 0.00010a |
| ***Gemmatimonadota*** | 0.05691 ± 0.00432^a^ | 0.02541 ± 0.00147^b^ | 0.00191 ± 0.00035^c^ | 0.00041 ± 0.00009c |
| ***Planctomycetota*** | 0.01311 ± 0.00159^c^ | 0.02291 ± 0.00080^c^ | 0.07601 ± 0.00639^b^ | 0.23451 ± 0.01604a |
| ***Verrucomicrobiota*** | 0.00251 ± 0.00034^c^ | 0.06051 ± 0.00612^b^ | 0.01301 ± 0.00092^a^ | 0.00251 ± 0.00031c |
| ***WPS-2*** | 0.00061 ± 0.00015^c^ | 0.00011 ± 0.00003^c^ | 0.01001 ± 0.00063^b^ | 0.02531 ± 0.00063a |
| ***Others*** | 0.04931 ± 0.00092^b^ | 0.06681 ± 0.00379^a^ | 0.03891 ± 0.00360^c^ | 0.02391 ± 0.00126d |

Different lowercase letters in the same row indicate significant differences between different treatments (*P* < 0.05).

HT: Bare tailing samples in cooper mine tailings; HB: BSCs samples in cooper mine tailings; LT: Bare tailing samples in rare earth mine tailings; LB: BSCs samples in rare earth mine tailings.

**Table S3** Relative abundances of major bacterial communities at order level of bare tailings and BSCs samples (Tukey’s HSD, P < 0.05).

| **Order** | **HT** | **HB** | **LT** | **LB** |
| --- | --- | --- | --- | --- |
| ***Bacillales*** | 0.00002 ± 0.00002^c^ | 0.00000 ± 0.00000^c^ | 0.00290 ± 0.00023^b^ | 0.07211 ± 0.00034^a^ |
| ***Burkholderiales*** | 0.24523 ± 0.00250^a^ | 0.23190 ± 0.00406^b^ | 0.01087 ± 0.00092^c^ | 0.00533 ± 0.00007^c^ |
| ***Caulobacterales*** | 0.00318 ± 0.00058^bc^ | 0.07621 ± 0.00252^a^ | 0.00078 ± 0.00016^c^ | 0.00535 ± 0.00036^b^ |
| ***Chitinophagales*** | 0.00786 ± 0.00035^c^ | 0.04340 ± 0.00292^b^ | 0.13214 ± 0.00538^a^ | 0.00008 ± 0.00005^c^ |
| ***Cytophagales*** | 0.00476 ± 0.00013^b^ | 0.10940 ± 0.00447^a^ | 0.00008 ± 0.00003^b^ | 0.00005 ± 0.00002^b^ |
| ***Elsterales*** | 0.00033 ± 0.00002^b^ | 0.00131 ± 0.00007^b^ | 0.07933 ± 0.00369^a^ | 0.00077 ± 0.00005^b^ |
| ***Gemmatimonadales*** | 0.05436 ± 0.00424^b^ | 0.02537 ± 0.00148^a^ | 0.00180 ± 0.00035^c^ | 0.00039 ± 0.00009^c^ |
| ***Geobacterales*** | 0.00084 ± 0.00019^b^ | 0.12875 ± 0.00742^a^ | 0.00002 ± 0.00002^b^ | 0.00000 ± 0.00000^b^ |
| ***Isosphaerales*** | 0.00096 ± 0.00011^c^ | 0.00001 ± 0.00001^c^ | 0.03165 ± 0.00304^b^ | 0.04404 ± 0.00132^a^ |
| ***Ktedonobacterales*** | 0.00000 ± 0.00000^c^ | 0.00000 ± 0.00000^c^ | 0.21379 ± 0.00596^b^ | 0.31550 ± 0.00150^a^ |
| ***Rhizobiales*** | 0.03802 ± 0.00080^a^ | 0.02022 ± 0.00117^c^ | 0.01334 ± 0.00069^d^ | 0.02770 ± 0.00133^b^ |
| ***Solirubrobacterales*** | 0.02768 ± 0.00019^b^ | 0.00021 ± 0.00007^d^ | 0.01158 ± 0.00010^c^ | 0.04923 ± 0.00249^a^ |
| ***Sphingomonadales*** | 0.11478 ± 0.00354^b^ | 0.04331 ± 0.00316^c^ | 0.00322 ± 0.00035^d^ | 0.01370 ± 0.00033^a^ |
| ***Subgroup_2*** | 0.00000 ± 0.00000^b^ | 0.00000 ± 0.00000^b^ | 0.07277 ± 0.00295^a^ | 0.00020 ± 0.00004^b^ |
| ***Tepidisphaerales*** | 0.00161 ± 0.00022^b^ | 0.00078 ± 0.00023^b^ | 0.00271 ± 0.00043^b^ | 0.18518 ± 0.01469^a^ |
| ***Xanthomonadales*** | 0.04780 ± 0.00152^a^ | 0.02793 ± 0.00075^b^ | 0.00052 ± 0.00007^c^ | 0.00016 ± 0.00007^c^ |
| ***Others*** | 0.45258 ± 0.00551^a^ | 0.29119 ± 0.00704^c^ | 0.42252 ± 0.00426^b^ | 0.28023 ± 0.01193^c^ |

Different lowercase letters in the row indicate significant differences between different treatments (*P* < 0.05).

HT: Bare tailing samples in cooper mine tailings; HB: BSCs samples in cooper mine tailings; LT: Bare tailing samples in rare earth mine tailings; LB: BSCs samples in rare earth mine tailings.

**Table S4** Relative abundances of major fungal communities at phylum level of bare tailings and BSCs samples (Tukey’s HSD, *P* < 0.05).

| **Phylum** | **HT** | **HB** | **LT** | **LB** |
| --- | --- | --- | --- | --- |
| ***Ascomycota*** | 0.71774 ± 0.02458^b^ | 0.70518 ± 0.00347^b^ | 0.98206 ± 0.00353^a^ | 0.94743 ± 0.00560^a^ |
| ***Basidiomycota*** | 0.20923 ± 0.02299^a^ | 0.04625 ± 0.00190^b^ | 0.00701 ± 0.00170^c^ | 0.00542 ± 0.00064^c^ |
| ***Chytridiomycota*** | 0.00000 ± 0.00000^b^ | 0.14182 ± 0.01270^a^ | 0.00000 ± 0.00000^b^ | 0.00001 ± 0.00001^b^ |
| ***Others*** | 0.07303 ± 0.00456^b^ | 0.10674 ± 0.01141^a^ | 0.01094 ± 0.00192^d^ | 0.04714 ± 0.00561^c^ |

Different lowercase letters in the same row indicate significant differences between different treatments (*P* < 0.05).

HT: Bare tailing samples in cooper mine tailings; HB: BSCs samples in cooper mine tailings; LT: Bare tailing samples in rare earth mine tailings; LB: BSCs samples in rare earth mine tailings.

**Table S5** Relative abundances of major fungal communities at order level of bare tailings and BSCs samples (Tukey’s HSD, P < 0.05).

| **Order** | **HT** | **HB** | **LT** | **LB** |
| --- | --- | --- | --- | --- |
| ***Chaetothyriales*** | 0.00097 ± 0.00005^c^ | 0.00166 ± 0.00020^c^ | 0.12870 ± 0.00478^b^ | 0.27070 ± 0.00670^a^ |
| ***Capnodiales*** | 0.02682 ± 0.00711^c^ | 0.01992 ± 0.00082^c^ | 0.03814 ± 0.00278^b^ | 0.27411 ± 0.01042^a^ |
| ***Pleosporales*** | 0.01127 ± 0.00270^b^ | 0.32504 ± 0.01017^a^ | 0.00680 ± 0.00099^c^ | 0.00721 ± 0.00117^c^ |
| ***Rhizophydiales*** | 0.00000 ± 0.00000^b^ | 0.14182 ± 0.01270^a^ | 0.00000 ± 0.00000^b^ | 0.00000 ± 0.00000^b^ |
| ***Saccharomycetales*** | 0.10143 ± 0.00786^a^ | 0.00000 ± 0.00000^b^ | 0.00078 ± 0.00050^b^ | 0.00002 ± 0.00001^b^ |
| ***Others*** | 0.85950 ± 0.00182^a^ | 0.51156 ± 0.02218^c^ | 0.82557 ± 0.00761^b^ | 0.44796 ± 0.00988^d^ |

Different lowercase letters in the same row indicate significant differences between different treatments (*P* < 0.05).

**Table S6** Parameters of microbial co-occurrence network.

| **Group** | **nodes** | **edges** | **connectance** | **average degree** | **global**  **clustering**  **coefficient** | **average**  **clustering**  **coefficient** | **average**  **neighborhood** | **centralization**  **degree** | **eigenvector**  **centralization** | **cohesion** | **modularity** | **Complexity** |
| --- | --- | --- | --- | --- | --- | --- | --- | --- | --- | --- | --- | --- |
| H_Bacteria | 689 | 126918 | 0.535 | 368.412 | 689 | 0.771 | 368.412 | 0.001 | 0.847 | 0.547 | 0.165 | 2.414 |
| L_Bacteria | 691 | 155826 | 0.654 | 451.016 | 691 | 0.845 | 451.016 | 0.001 | 0.923 | 0.574 | 0.101 | 2.898 |
| H_Fungi | 268 | 1721 | 0.048 | 12.843 | 40 | 0.980 | 12.843 | 0.000 | 0.054 | 0.987 | 0.846 | 0.280 |
| L_Fungi | 276 | 6195 | 0.163 | 44.891 | 222 | 0.753 | 44.891 | 0.004 | 0.358 | 0.957 | 0.481 | 0.425 |

H: Copper mine tailings samples

L: Rare earth mine tailings samples
